# Mechanisms for controlling Dorsal nuclear levels

**DOI:** 10.3389/fcell.2024.1436369

**Published:** 2024-08-05

**Authors:** James McGehee, Angelike Stathopoulos

**Affiliations:** California Institute of Technology, Division of Biology and Biological Engineering, Pasadena, CA, United States

**Keywords:** *Drosophila melanogaster*, Dorsal transcription factor, nuclear import and export, shuttling, nuclear spacing, phosphorylation, sumoylation, post-translational modification

## Abstract

Formation of the Dorsal nuclear-cytoplasmic gradient is important for the proper establishment of gene expression patterns along the dorsal-ventral (DV) axis during embryogenesis in *Drosophila melanogaster*. Correct patterning of the DV axis leads to formation of the presumptive mesoderm, neurogenic ectoderm, dorsal ectoderm, and amnioserosa, which are tissues necessary for embryo viability. While Toll signaling is necessary for Dorsal gradient formation, a gradient still forms in the absence of Toll, suggesting there are additional mechanisms required to achieve correct nuclear Dorsal levels. Potential mechanisms include post-translational modification, shuttling, and nuclear spacing. Post-translational modification could affect import and export rates either directly through modification of a nuclear localization sequence or nuclear export sequence, or indirectly by affecting interactions with binding partners that alter import and export rates. Shuttling, which refers to the facilitated diffusion of Dorsal through its interaction with its cytoplasmic inhibitor Cactus, could regulate nuclear levels by delivering more Dorsal ventrally. Finally, nuclear spacing could result in higher nuclear levels by leaving fewer nuclei in the ventral domain to uptake Dorsal. This review details how each of these mechanisms may help establish Dorsal nuclear levels in the early fly embryo, which serves as a paradigm for understanding how the dynamics of graded inputs can influence patterning and target gene expression. Furthermore, careful analysis of nuclear Dorsal levels is likely to provide general insights as recent studies have suggested that the regulation of nuclear import affects the timing of gene expression at the maternal-to-zygotic transition.

## 1 An introduction to formation of the Dorsal gradient

Over the course of embryogenesis, patterning of gene expression and differentiation give rise to the tissues that ultimately make up an organism. In the fruit fly *Drosophila melanogaster*, two morphogens, Bicoid and Dorsal, pattern the embryo along the anterior-posterior (AP) and dorsal-ventral (DV) axis, respectively (Nüsslein-Volhard et al., 1980; Anderson and Nüsslein-Volhard, 1984; Driever and Nüsslein-Volhard, 1988a; Driever and Nüsslein-Volhard, 1988b). Bicoid forms a gradient of mRNA and protein in the anterior (Driever and Nüsslein-Volhard, 1988a), while Dorsal protein forms a nuclear concentration gradient with the highest nuclear Dorsal levels on the ventral side of the embryo (Steward et al., 1988; Roth et al., 1989; Steward, 1989).

The process of forming the Dorsal gradient is regulated by Toll signaling (Steward et al., 1988; Roth et al., 1989; Steward, 1989), which occurs early in embryogenesis, stages 3–5 (Hashimoto et al., 1991; Anderson et al., 1985; Hashimoto et al., 1988), and culminates just prior to gastrulation (DeLotto et al., 2007; Reeves et al., 2012). Once Toll signaling is activated, Dorsal is released from its inhibitor Cactus, which sequesters Dorsal in the cytoplasm. Specifically, Cactus is phosphorylated and degraded, freeing Dorsal to enter the nucleus (Belvin et al., 1995). This is achieved through the action of Pelle kinase, which phosphorylates itself (Shen and Manley, 1998; Shen and Manley, 2002; Towb et al., 2001), the adaptor protein Tube (Grosshans et al., 1994; Towb et al., 2001), Toll (Shen and Manley, 1998), and potentially Cactus (Grosshans et al., 1994; Reach et al., 1996; Daigneault et al., 2013). While degradation of Cactus is necessary to allow the nuclear accumulation of Dorsal, a shallow, Toll-dependent gradient of Dorsal still forms in embryos that lack Cactus (Roth et al., 1991; Bergmann et al., 1996). This suggests that Toll regulates Dorsal in a Cactus-independent manner, potentially through phosphorylation.

Nuclear localization is necessary for Dorsal, a transcription factor, to control target gene expression in a concentration dependent manner (Roth et al., 1989; Stathopoulos et al., 2002; Reeves et al., 2012). High levels of Dorsal on the ventral side are required for expression of genes in the presumptive mesoderm, such as *snail* (*sna*) and *twist* (*twi*). Intermediate levels of Dorsal activate genes in the lateral region, including *short gastrulation* (*sog*), *ventral neuroblasts defective* (*vnd*), *intermediate neuroblasts defective* (*ind*), and *brinker* (*brk*) (Reeves et al., 2012). In addition to its role as an activator, these high and intermediate levels of Dorsal are able to repress genes that should only be expressed in the dorsal region, such as *zerknüllt* (*zen*) and *decapentaplegic* (*dpp*) (Kirov et al., 1993). Wildtype Dorsal levels and distribution are important for several early developmental processes. In the absence of Dorsal, the cuticle adopts a twisted morphology, where only dorsal cuticle is present (Roth et al., 1989). Dorsal is also necessary for gastrulation through its activation of *twi* and *sna*, since a loss of both results in failure of presumptive mesoderm invagination (Leptin and Grunewald, 1990). Finally, the neurogenic ectoderm also fails to form in the absence of Dorsal (Roth et al., 1989).

In addition to Toll-dependent Cactus degradation, other factors may regulate nuclear Dorsal levels. These include post-translational modifications, which could affect nuclear import or export rates (Norris and Manley, 1992; Whalen and Steward, 1993; Gillespie and Wasserman, 1994; Drier et al., 1999); shuttling, which can concentrate Dorsal protein ventrally (Carrell et al., 2017); and nuclear spacing, which is thought to affect nuclear concentration by moving nuclei dorsally (Xue et al., 2023). These mechanisms for controlling Dorsal nuclear levels are not mutually exclusive.

In addition to these, there are other potential mechanisms that may regulate Dorsal, including the role of Toll signaling and Cactus inhibition. Since these mechanisms have been reviewed before (Stein and Stevens, 2014; Schloop et al., 2020a), we will not cover them in this review. Other potential protein interactions that may play an important role in determining the nuclear concentration of Dorsal include Tamo, a nuclear import regulator shown to interact with Dorsal (Minakhina et al., 2003); CRM1, a nuclear export factor shown to be important for export of Dorsal when it is blocked by drug treatment (Xylourgidis et al., 2006; DeLotto et al., 2007); and WntD, an inhibitor of Dorsal that has been shown to affect *twi* and *sna* expression (Ganguly et al., 2005; Rahimi et al., 2019). Also, Dorsal is thought to act synergistically with the transcription factors Twi and Zelda to activate target gene expression (Ip et al., 1992; Shirokawa and Courey, 1997; Yamada et al., 2019). Below, we focus our review on how post-translational modifications, shuttling, and nuclear spacing lead to correct nuclear Dorsal levels, as these mechanisms are likely to explain how to fine-tune the nuclear concentration of Dorsal and affect the correct patterning of target genes.

## 2 Post-translational modification of Dorsal

**FIGURE 1:**
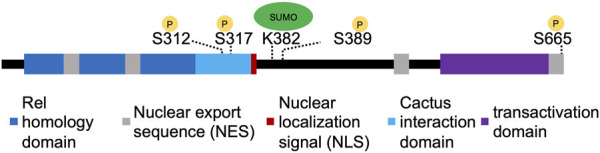
Organization of Dorsal protein domains. The different domains of Dorsal, with experimentally confirmed sites of phosphorylation or SUMOylation indicated with yellow or green circles, respectively.

One mechanism that could refine Dorsal nuclear distribution is post-translational modifications, such as phosphorylation, that affect nuclear import or export. Dorsal, as well as its mammalian homolog NF-κB, have been shown to be phosphorylated on multiple residues (Norris and Manley, 1992; Whalen and Steward, 1993; Gillespie and Wasserman, 1994; Drier et al., 1999; Viatour et al., 2005). Drier et al. (1999) observed a loss of Dorsal nuclear import upon mutating six serine residues to alanine: S70, S79, S103, S213, S312, and S317. While the authors argued that only mutation of S317 affects the distribution of phosphorylated forms, to our knowledge, these sites have not been confirmed to be phosphorylated by mass spectrometry. The only phosphorylation sites that were detected in a system-wide assay of phosphorylation were S389 and S665 (Hilger et al., 2009). One of these serine residues, S665, is located close to or within Dorsal’s nuclear export sequence (NES) (Figure 1) (Xylourgidis et al., 2006; DeLotto et al., 2007). Since the phosphorylation site is close to a NES, phosphorylation could conceivably control the export rate and thus nuclear Dorsal levels.

Additionally, removing Cactus does not lead to uniform Dorsal nuclear levels around the embryo circumference (Roth et al., 1991; Bergmann et al., 1996). One possible explanation for this is that Toll signaling may also phosphorylate Dorsal. In this model, Toll signaling degrades Cactus so Dorsal is free to enter the nucleus, and reduces Dorsal’s nuclear export rate by phosphorylating the NES.

Alternatively, Dorsal could be phosphorylated by other factors, either in addition to or instead of Toll signaling. For example, Dorsal has been reported to be phosphorylated at serine residue 312 near the NLS by the c-AMP dependent protein kinase (PKA), and this modification was found to be important for regulating import through interaction with importin in cell culture (Briggs et al., 1998). However, others were unable to detect this particular phosphorylation (Drier et al., 1999). Instead, Drier et al. (1999) hypothesized that S312 may control Dorsal stability *in vivo* (Drier et al., 1999). Another kinase recently shown to affect Dorsal nuclear localization is Raf kinase, a MAP3K (Lusk et al., 2022). Lusk et al. (2022) found a strong dorsalizing phenotype when they screened a new library of mutations, which mapped to the gene encoding Raf kinase. They then observed that Dorsal is cytoplasmic in this mutant and Dorsal targets like *twist* are expressed at low levels (Lusk et al., 2022). This raises the possibility that Raf could participate directly in phosphorylating Dorsal or Cactus, although it cannot be ruled out that Raf is involved elsewhere in the pathway. Regardless of the source of phosphorylation, these modifications could be constitutive, or could be modulated by downstream events of Dorsal activation to further increase or decrease nuclear Dorsal levels in different contexts.

In addition to the action of kinases, dephosphorylation can also modulate nuclear import and export. Cactus has been found to be phosphorylated and degraded in response to calcium (Liu et al., 1997). However, calcium has the opposite effect on Dorsal, leading to dephosphorylation (Kubota and Gay, 1995). While the effects of these phosphorylation and dephosphorylation events are unknown, they could be used to regulate levels of Dorsal or localization. One can imagine that if phosphorylation is used to fine tune Dorsal levels, then dephosphorylation may be an important mechanism to reverse these changes.

Dorsal has also been shown to be SUMOylated by Ubc9, a SUMO conjugating enzyme (Bhaskar et al., 2000). Bhaskar et al. (2002) identified K382 as the site of SUMOylation (Figure 1), and found that SUMOylation played an important role in cells and larvae’s ability to activate antimicrobial genes. Hedge et al. (2022) generated a CRISPR mutant (Dorsal^K382R^) that prevents SUMOylation and assayed it during embryogenesis. They found that development proceeds normally in this mutant but that Dorsal^K382R^ was able to more strongly activate transcription of its targets in conditions of haploinsufficiency. This suggests that SUMOylation may serve as part of a negative feedback loop to modulate downstream effects (Hegde et al., 2022). The results from these studies suggest that the regulation of Dorsal through SUMOylation may be context specific. It is also possible that SUMOylation of Dorsal triggers its nuclear export as has been demonstrated for the *Drosophila* transcription factor Medea although this has not yet been observed (Miles et al., 2008). SUMOylation may be an additional mechanism used instead of or in conjunction with other post-translational modifications, such as phosphorylation, to control Dorsal activity.

**FIGURE 2:**
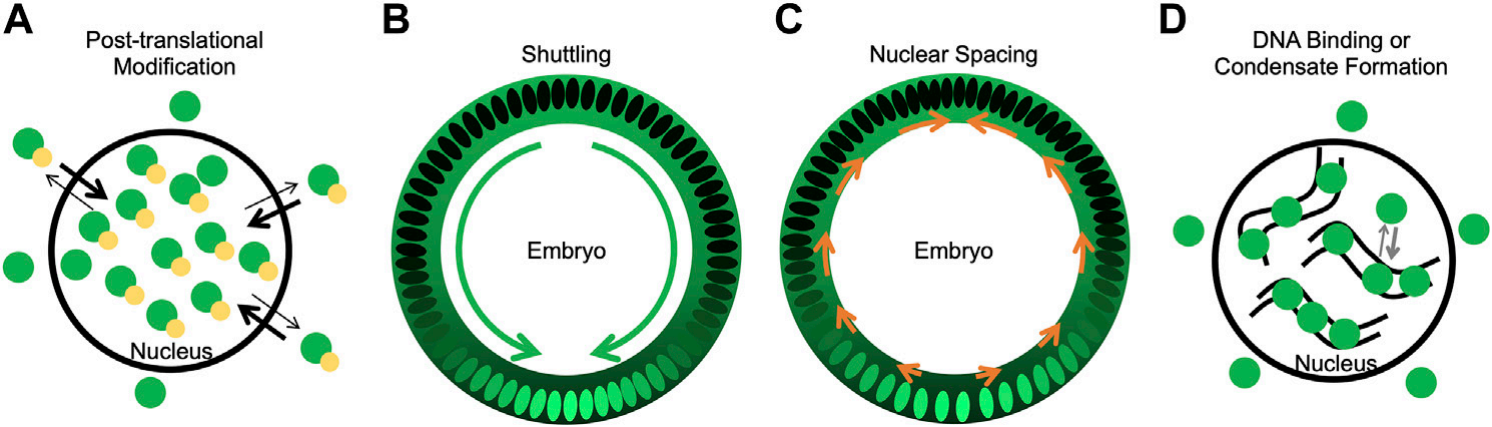
Mechanisms potentially contributing to Dorsal nuclear levels. **(A)** A model of how phosphorylation could bias import/export rates such that phosphorylated Dorsal is nuclear. Dorsal is in green, phosphorylation in yellow. **(B)** A model of shuttling, where Dorsal-Cactus diffuses towards the ventral midline as Toll signaling degrades Cactus and Dorsal enters ventral and lateral nuclei. Dorsal concentration both in the nucleus and cytoplasm is shown in green. Green arrows represent the flow of Dorsal-Cactus. **(C)** A model of how changes in nuclear spacing, where nuclei move more dorsally, could leave fewer nuclei on the ventral side which uptake the cytoplasmic Dorsal. Orange arrows represent the movement of nuclei towards the dorsal side. **(D)** A model of how DNA binding or condensate formation may reduce the pool of Dorsal available for export, thus allowing Dorsal to accumulate in nuclei. Curved black lines represent DNA, and Dorsal is in green.

Post-translational modifications, either through phosphorylation, dephosphorylation, or SUMOylation, likely play an important role in determining nuclear Dorsal levels. These modifications can have different effects including regulating stability, protein-protein interactions, nuclear import/export, and target gene expression and could function to fine-tune nuclear Dorsal levels in specific contexts (Figure 2A).

## 3 Shuttling

Shuttling, or the facilitated diffusion of proteins in a tissue, represents another mechanism that could affect the nuclear distribution of Dorsal. Toll signaling degrades Cactus ventrally, resulting in a concentration gradient of Dorsal-Cactus. Since Dorsal-Cactus would be highest on the dorsal side of the embryo, Dorsal-Cactus complexes will diffuse ventrally (Carrell et al., 2017). In this model, the nuclei act as sinks, preventing free Dorsal from diffusing away from the ventral side of the embryo (Figure 2B). Four features of the Dorsal-Cactus system support a shuttling mechanism: 1) Dorsal binds to Cactus, which acts as a carrier molecule; 2) the Dorsal-Cactus complex can diffuse; 3) the complex is degraded in a spatially-dependent manner, specifically, in ventral regions where Toll signaling is active; and 4) upon signaling activation, once Dorsal is free of its carrier, Cactus, the Dorsal molecule is captured by the nucleus or degraded (Schloop et al., 2020b). Any system with these four features balanced in the proper way would support shuttling.

To test this shuttling model, Carrell et al. (2017) fused a photoactivatable green fluorescent protein to Dorsal (Dorsal-paGFP) to determine how far molecules could diffuse. Dorsal-paGFP was activated in a small window and the distribution of the fluorescent signal was tracked over time. They found that on the ventral side, Dorsal-paGFP signal was only detected in ∼6–7 nuclei surrounding the area of activation, whereas on the dorsal side, Dorsal-paGFP signal was detected in the entire field of view after 90 min (Carrell et al., 2017). This finding supports the model that Dorsal-Cactus can shuttle towards the ventral side from the dorsal side, but diffusion of free Dorsal away from the ventral side is restricted. To further test how diffusion rates may play a role in determining nuclear concentration, the authors also decreased the mobility of Dorsal by attaching monomeric GFP (mGFP) and dimeric GFP (dGFP), respectively. They found that the decreased mobility of the larger fusion proteins was associated with an increase in Dorsal gradient width and a decrease in peak Dorsal levels in ventral nuclei (Carrell et al., 2017). These findings suggest that shuttling is an important mechanism both for defining the gradient boundaries and for controlling the nuclear concentration of Dorsal.

## 4 Nuclear spacing

Dorsal nuclear concentration could also be affected by the density and distribution of nuclei around the circumference of the embryo (Figure 2C). One can imagine that, if the concentration of cytoplasmic Dorsal is uniform around the embryo but nuclei spacing is less dense on the ventral side, this could increase Dorsal nuclear levels ventrally as more Dorsal would be imported into fewer nuclei. Indeed, it has been shown that nuclei are denser on the dorsal side of the embryo than the ventral side of the embryo (Xue et al., 2023). Xue et al. (2023) determined that Dorsal and Decapentaplegic (Dpp), the *Drosophila* homolog of BMP, are important regulators of this change in nuclei density. Dorsal regulation is known to constrain *dpp* expression to the dorsal side of the embryo, where Dpp acts as a ligand to activate BMP signaling and produce pMad (Ferguson and Anderson, 1992a; Ferguson and Anderson, 1992b; Kirov et al., 1993). Xue et al. (2023) determined that this Dpp/BMP signaling was primarily responsible for the dorsal-ward movement of nuclei.

The authors also identified the downstream effectors of this process, encoded by *frazzled* and *GUK-holder*, and noted a change in expression of DV markers when these genes are mutated (Xue et al., 2023). They determined that these changes occurred as a result of altering the Dorsal gradient. Specifically, the gradient was wider and peak Dorsal protein levels were reduced at the ventral midline. They suggest that these changes in the Dorsal gradient are a direct result from the loss of cell movements mediated by *frazzled* and *GUK-holder*. They propose a model where Dorsal and Dpp cause nuclei density to change, which feeds back into proper gradient formation and target gene expression (Xue et al., 2023). While this study does not rule out additional direct or indirect roles for these factors downstream of Dorsal or Dpp, the changes to the Dorsal and Dpp gradient correspond to observed changes in gene expression. This suggests that nuclear spacing plays an important role in modulating nuclear Dorsal levels.

## 5 Summary and unanswered questions regarding the control of nuclear Dorsal levels

While each of these proposed mechanisms could individually determine final Dorsal nuclear levels, they are not mutually exclusive and could work together. For example, the nuclear import/export rate could be fixed or it could be modulated dynamically to increase or decrease Dorsal levels. Shuttling could move more Dorsal into the ventral domain of the embryo, increasing the amount of Dorsal available to be imported into nuclei. Finally, nuclear spacing could result in fewer nuclei present ventrally to uptake Dorsal, causing an increase in nuclear Dorsal levels ventrally. Regardless, the import/export rate is fundamentally important for all of these mechanisms, as Dorsal must be able to enter the nucleus to act as a transcription factor and affect downstream targets.

It has been shown that timing of nuclear import may be an important mechanism for controlling the time of gene activation in *Xenopus laevis* (Nguyen et al., 2022). Nguyen et al. (2022) found that the timing of proteins entering the nucleus varied widely, and that the timing of transcription factor import and the onset of gene transcription were strongly correlated. In addition, the importin affinities of various proteins also correlated with the timing of their nuclear import. Their findings suggest a model where importin affinity controls the timing of nuclear import and subsequent gene transcription during early embryonic development.

In addition to the mechanisms reviewed here, there could be other important mechanisms that play a role in correct Dorsal gradient formation, including DNA binding and condensate formation (Figure 2D). DNA binding could potentially result in a lower pool of Dorsal being available to interact with the export machinery. Similarly, condensates could play a key role in proper formation of the gradient by sequestering Dorsal and reducing the Dorsal that is able to interact with export machinery. While some condensates form through liquid-liquid phase separation (LLPS) and are concentration-dependent, other types of condensates or membraneless compartments form through multivalent biomolecular interactions (Mittag and Pappu, 2022). These types of condensates could potentially sequester proteins and affect their biochemical reactions (Banani et al., 2017; Alberti and Dormann, 2019; Chong et al., 2022) including, but not yet demonstrated for, nuclear export. Future studies are needed to show if condensates can sequester proteins from export. Dorsal has been shown to increase in local concentration at the site of transcription, forming hubs (Yamada et al., 2019; Hamamoto et al., 2023), but whether or not these hubs are condensates is currently unknown (Palacio and Taatjes, 2022). How Dorsal condensates control its nuclear levels remains to be determined.

Perturbations to any of these mechanisms that affect Dorsal nuclear levels are likely to have direct effects on target gene expression and embryo viability. While it has not been shown whether post-translational modifications such as phosphorylation affect target gene expression directly, mutating S389 or S665 in Dorsal will likely elucidate the role of phosphorylation in these processes. Shuttling has been shown to play a role in specifying the width of the *sna* expression domain and when shuttling is disrupted *sna* expression is narrower than wildtype (Carrell et al., 2017). Future studies are needed to determine if intermediate target genes are affected. Finally, mutation of *fra*, which encodes an effector involved in nuclear spacing, was already demonstrated to exhibit changes in expression of six DV target genes regulated by Dorsal or Dpp including *vnd* and *sna* (Xue et al., 2023).

These fine tuning mechanisms likely provide a robust response to perturbations (genetic, environmental, stochastic etc.) such that proper patterning occurs and embryo viability is maintained. One can imagine that disrupting one of these mechanisms for fine-tuning Dorsal levels may only result in a small reduction in viability but disrupting multiple mechanisms at once could lead to inviability. Thus, fine tuning nuclear Dorsal levels through post-transcriptional modification, shuttling, and nuclear spacing likely plays an important role in the viability of developing embryos by potentially positioning boundaries of gene expression domains but other regulatory mechanisms including DNA binding and condensate formation may contribute as well.
